# Supplementary material for: Ultrathin Single-Walled Carbon Nanotube Surface Wave Absorbers for Terahertz Dielectric Waveguides
Source: Nat Commun. 2025 Dec 2;16:10868. doi: 10.1038/s41467-025-66559-1 (PMC12675539; doi:10.1038/s41467-025-66559-1)
Supplement: Supplementary file 1 — Supplementary Information [file 41467_2025_66559_MOESM1_ESM.pdf]

Supplementary Information for: Ultrathin Single-Walled Carbon Nanotube Surface Wave Absorbers for Terahertz Dielectric Waveguides

Nikolaos Xenidis, Mehrdad Rezaei Golghand, Nikita I. Raginov, Joachim Oberhammer, Dmitry V. Krasnikov, Albert G. Nasibulin, Dmitry V. Lioubtchenko

I. Supplementary Note 1: Experimental

The single-walled carbon nanotube (SWCNT) films used in this study are shown in Supplementary Fig. 1b; a SWCNT-coated dielectric rod waveguide (DRW) post dry-transfer is shown in Supplementary Fig. 1c, with its top broad wall covered by a SWCNT layer. A schematic representation of the measurement setup is shown in Supplementary Fig. 1a, with the coupling spikes of the loaded DRW inserted inside the hollow metallic WR-5.1 (140-220 GHz) waveguides, while the actual measurement setup in the lab is shown in Supplementary Fig. 1d.

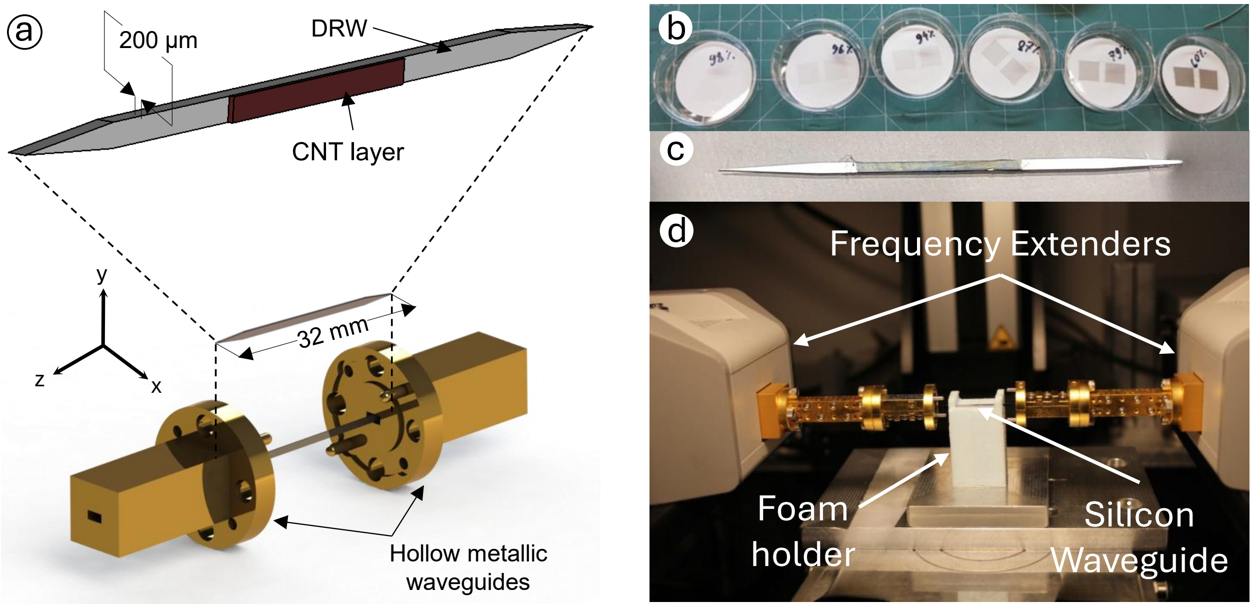

Supplementary Fig. 1 Experimental setup and samples for THz measurements. **a** Schematic of the THz measurement setup, using a tapered silicon waveguide placed between the hollow metallic waveguides. **b** Deposited SWCNT films on the polymer substrate with various optical transmittance values ranging from 60% to 98% (thicknesses 2 - 53 nm). **c** Silicon waveguide with SWCNT film on it. **d** Actual measurement setup with the WR-5.1 extenders at the 140-220 GHz frequency range.
